# Periodontitis-Induced Immune Reprogramming: Implications for Cancer Immunotherapy Response

**DOI:** 10.3390/biomedicines14020480

**Published:** 2026-02-22

**Authors:** Claudia Florina Bogdan-Andreescu, Ștefan-Dimitrie Albu, Dan Alexandru Slăvescu, Lucia Bubulac, Viorica Tudor, Oana Botoacă, Andreea-Mariana Bănățeanu, Emin Cadar, Cristina-Crenguţa Albu

**Affiliations:** 1 Department of Speciality Disciplines, Faculty of Dental Medicine, “Titu Maiorescu” University, 031593 Bucharest, Romania; claudia.andreescu@prof.utm.ro (C.F.B.-A.); oana.botoaca@prof.utm.ro (O.B.); andreea.banateanu@prof.utm.ro (A.-M.B.); 2 Department of Periodontology, Faculty of Dentistry, “Carol Davila” University of Medicine and Pharmacy, 020021 Bucharest, Romania; stefan.albu@umfcd.ro; 3 Department of Dentistry, Faculty of Medicine and Pharmacy, University of Oradea, 410073 Oradea, Romania; 4 Department of Family Medicine III, Faculty of Medicine, “Carol Davila” University of Medicine and Pharmacy, 050474 Bucharest, Romania; 5 Integrated Outpatient Department, “Prof. Dr. Agrippa Ionescu” Clinical Emergency Hospital, 011356 Bucharest, Romania; tudorviorica_med@yahoo.com; 6 Faculty of Pharmacy, “Ovidius” University, 900470 Constanţa, Romania; emin.cadar@365.univ-ovidius.ro; 7 Department of Genetics, Faculty of Dentistry, “Carol Davila” University of Medicine and Pharmacy, 020021 Bucharest, Romania; cristina.albu@umfcd.ro

**Keywords:** periodontitis, immunotherapy, immune reprogramming, PD-1, PD-L1, macrophage polarization, T-cell exhaustion, NF-κB, STAT3, oral–systemic axis, cancer immunology

## Abstract

**Background:** Chronic periodontitis is a prevalent inflammatory disease. It goes beyond the oral cavity, exerting systemic immunomodulatory effects through continuous low-grade inflammation, microbial dysbiosis, and cytokine spillover. Accumulating evidence suggests that the immunological consequences of periodontitis may influence systemic immune homeostasis and alter responses to cancer immunotherapies, specifically checkpoint blockade. **Objectives**: This narrative review describes how periodontal inflammation induces systemic immune reprogramming. It also investigates possible effects on the efficacy of immunotherapy. **Methods**: The paper synthesizes current findings on molecular and cellular mechanisms linking periodontitis to immune dysfunction. It underscores the mutual signaling pathways NF-κB, STAT3, and PD-1/PD-L1 that connect oral and systemic immunity. **Results**: Chronic periodontal inflammation reprograms innate and adaptive immune responses. It elevates proinflammatory mediators, such as IL-1β, IL-6, and TNF-α. It alters T-cell polarization and promotes myeloid cell “training”. This process may lead to immune exhaustion, impaired antigen presentation, and treatment resistance. Preclinical and new clinical data suggest that controlling periodontal inflammation may partially reduce systemic inflammatory burden, although clinical evidence in immunotherapy-treated cancer cohorts remains limited. **Conclusions**: Periodontal health should be considered in the management of immunotherapy. This can facilitate new studies that integrate oral and systemic immunology. Understanding the two-way link between periodontal inflammation and systemic immune reprogramming may offer fresh opportunities for personalized immunomodulation and combined interventions.

## 1. Introduction

Periodontitis is a highly prevalent, chronic inflammatory disease resulting from pathogenic shifts in the oral microbiome, and it has traditionally been regarded as a localized oral condition [1,2]. However, recent evidence demonstrates that its effects are not limited to the gingival tissues. Instead, chronic periodontal inflammation promotes persistent low-grade systemic inflammation, facilitates the dissemination of microbial components, and induces sustained immune activation [3,4,5,6]. These perturbations alter circulating myeloid and lymphoid cells, disrupt cytokine networks, and reshape overall immune homeostasis [7,8,9,10,11]. This form of systemic immune reprogramming has now been recognized as a contributing factor for multiple chronic conditions, including cardiovascular disease, metabolic dysfunction, respiratory conditions, musculoskeletal disorders, reproductive system issues, and multiple malignancies [12,13,14,15,16,17,18].

At the same time, cancer immunotherapy, particularly immune checkpoint inhibitors (ICIs), has transformed oncology by using endogenous antitumor immunity [19,20,21,22]. However, only some patients achieve lasting responses, and the reasons for success or resistance are still not fully understood [23,24,25]. Resistance can result from multiple factors, including the tumor microenvironment (TME), genetic and epigenetic alterations, and tumor-driven protective or regenerative programs that support inflammation and related mechanisms [26,27,28,29]. While tumor-intrinsic features and the gut microbiome have been extensively investigated, the possible influence of oral inflammatory disease on immunotherapy outcomes has received comparatively little attention [30,31,32]. Importantly, periodontal pathogens and the inflammatory environment they sustain can promote T-cell exhaustion, expand immunosuppressive myeloid populations, and upregulate immune checkpoint pathways such as PD-1/PD-L1 (programmed cell death-1/ programmed death ligand-1)—molecular routes directly relevant to ICI efficacy [33,34,35,36].

Emerging preclinical and clinical observations suggest that periodontitis may shape systemic immune tone in ways that either hinder or recalibrate antitumor immune responses [34,37,38,39]. This potential introduces a persuasive question: Could chronic periodontal inflammation represent a previously unrecognized—and potentially modifiable—determinant of cancer immunotherapy response? Filling this void has the potential to advance our knowledge about host–microbiome–immune interactions as well as to identify an accessible, clinically actionable target for improving treatment outcomes.

The objectives of this review are to define the key mechanisms by which chronic periodontitis induces systemic immune reprogramming; to examine how these immune alterations intersect with pathways controlling immune checkpoint blockade; and to evaluate whether periodontal inflammation represents an underrecognized, yet potentially modifiable, host factor influencing cancer immunotherapy. Ultimately, this review addresses the main research question: Can chronic periodontal disease shape systemic immune fitness in ways that impact cancer immunotherapy outcomes?

This paper synthesizes current knowledge on the systemic immunological consequences of periodontitis, resulting from the axis of oral inflammation and systemic inflammation, and examines their possible consequences for cancer immunotherapy responses. By bringing together perspectives from microbiology, immunology, genetics, and oncologic therapeutics, we aim to draw attention to an underrecognized axis of host–oral microbiome immune modulation. In doing so, we propose a conceptual framework to guide future mechanistic investigations and translational studies that may ultimately shape plans to optimize immunotherapy outcomes.

Here, we propose that chronic periodontitis acts as a systemic immune-conditioning factor capable of reshaping innate and adaptive immune responsiveness through persistent inflammatory signaling and durable epigenetic reprogramming. We argue that this altered immune set point may predispose to immune exhaustion and tolerance, thereby influencing responsiveness to immune checkpoint blockade. By integrating oral immunology with cancer immunotherapy, this review positions periodontal inflammation as a previously underappreciated host determinant of immunotherapy outcomes.

This narrative review was conducted using a structured literature search of PubMed/MEDLINE, Scopus, Web of Science and Google Scholar databases through December 2025. The search strategy combined the following terms: “periodontitis”, “immune reprogramming”, “trained immunity”, “systemic inflammation”, “tumor microenvironment”, “immune checkpoint blockade”, “PD-1/PD-L1”, and “cancer immunotherapy”. Article selection was based on relevance to the associations among periodontal inflammation, systemic immune modulation, and immunotherapy response. Recent high-impact clinical and translational studies, and evidence supporting biologically plausible immune pathways were prioritized. Due to the narrative nature of this review, formal systematic screening and meta-analysis were not performed. All efforts were made to provide a balanced and comprehensive synthesis of the available evidence.

In this review, the term immune reprogramming is used to describe persistent functional changes in immune cells induced by chronic inflammatory conditions [40]. Immune reprogramming may involve both innate and adaptive compartments. Trained immunity refers specifically to memory-like responses in innate immune cells (monocytes, macrophages, NK cells) driven by epigenetic and metabolic rewiring following microbial or inflammatory stimulation, often resulting in heightened secondary inflammatory responses [41]. In contrast, adaptive immune exhaustion is a distinct phenomenon characterized by progressive functional impairment of T cells during chronic antigen exposure, frequently accompanied by upregulation of inhibitory receptors such as PD-1 [42]. Distinguishing between these processes is essential, as trained immunity may amplify systemic inflammation, whereas adaptive exhaustion may contribute to immunosuppression within tumor settings.

## 2. Mechanisms of Systemic Immune Reprogramming in Periodontitis

### 2.1. Cytokine Spillover and Chronic Inflammation

**Figure 1:**
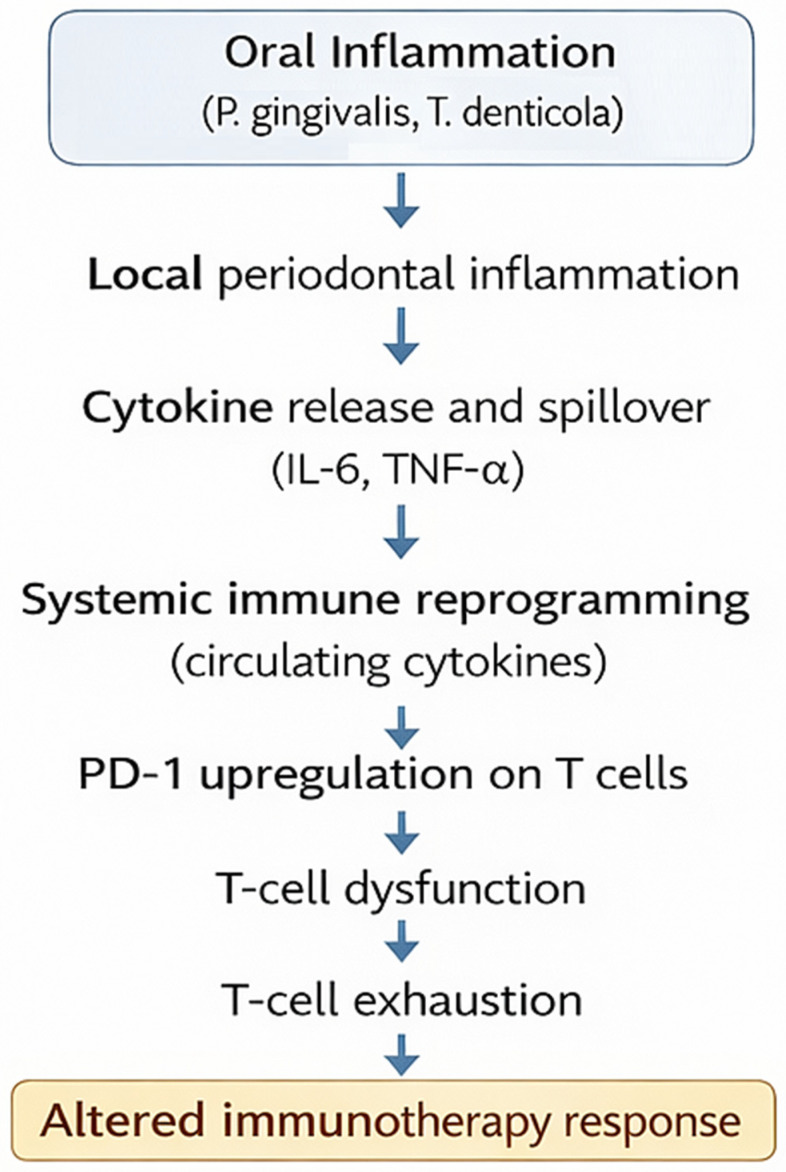
Mechanisms of systemic immune reprogramming in periodontitis. Legend: Overview of the cascade from local periodontal inflammation and cytokine spillover (IL-6, TNF-α) to systemic immune reprogramming characterized by PD-1 upregulation, T-cell dysfunction, and exhaustion. These processes establish persistent systemic immune alterations and may modulate responses to cancer immunotherapy.

The systemic consequences of periodontitis extend far beyond the oral cavity and can disturb immune homeostasis by persistently releasing inflammatory mediators into circulation. This process, known as cytokine spillover, represents the first step of systemic immune reprogramming [43,44]. Local production of proinflammatory cytokines and acute-phase reactants translates into measurable systemic alterations that sustain chronic low-grade inflammation [45]. The following subsections delineate the sequential cascade—from elevated cytokine levels and their vascular dissemination to the downstream activation and exhaustion of immune and endothelial compartments—that collectively shape the systemic inflammatory milieu characteristic of periodontitis. Understanding these mechanisms provides a base for clarifying how local periodontal inflammation evolves into a systemic immunoinflammatory state. Figure 1 provides an integrated framework linking chronic periodontal inflammation with systemic immune remodeling and the potential modulation of cancer immunotherapy efficacy.

#### 2.1.1. Elevated IL-1β, IL-6, TNF-α, and CRP in Chronic Periodontitis

Periodontitis is a classic example of a localized chronic infection capable of eliciting systemic immunoinflammatory consequences. The condition is not confined to periodontal tissues but acts as a systemic inflammatory driver, mediated mainly through the spillover of cytokines and acute-phase proteins into the bloodstream. High blood concentrations of interleukin-1β (IL-1β), interleukin-6 (IL-6), tumor necrosis factor-alpha (TNF-α), and C-reactive protein (CRP) are a constant finding in individuals with chronic periodontitis compared with healthy controls [46]. These mediators serve not only as biomarkers of local inflammation but also as potent effectors in systemic immune reprogramming. A comprehensive meta-analysis and spline-based meta-regression confirmed that circulating levels of IL-1β, IL-6, and TNF-α are significantly elevated in periodontitis and decrease only partially after intensive non-surgical periodontal therapy [47]. Similar temporal trends have been reported, showing transient cytokine surges after mechanical debridement followed by gradual normalization [48]. These findings indicate that even a temporary periodontal inflammatory episode contributes to measurable systemic cytokine fluctuations, maintaining a low-grade and persistent systemic inflammation [48]. Among these mediators, IL-6 acts as a dual-function cytokine, directing both local inflammation and systemic acute-phase responses through hepatic signaling that induces CRP and fibrinogen synthesis [49,50]. IL-6, IL-1β, and TNF-α have been emphasized as reliable diagnostic and prognostic biomarkers across chronic inflammatory diseases, including periodontitis [18]. Supplementary data showed that increased IL-6 levels are directly correlated with alveolar bone loss and disease severity [49]. Meta-analyses on CRP further support the systemic impact of periodontal inflammation [49]. It has been demonstrated that CRP levels are consistently higher in patients with periodontitis and decrease following periodontal therapy, suggesting a causal association between oral and systemic inflammation [51].

#### 2.1.2. Dissemination of Inflammatory Mediators via Systemic Circulation

This persistent inflammatory state is sustained through systemic dissemination of locally generated mediators. Locally, cytokines (IL-1β, IL-6, TNF-α) and prostaglandin E2 diffuse from periodontal tissues into the circulation, transforming a localized lesion into a systemic inflammatory trigger [38,46]. Epithelial ulceration within periodontal pockets facilitates leakage of cytokines and bacterial components into blood vessels, where they engage circulating leukocytes and endothelial cells [52]. At the molecular level, this dissemination is governed mainly by TLR4 (toll-like receptor) engagement by *Porphyromonas gingivalis* lipopolysaccharide, which activates NF-κB (nuclear factor kappa-B) signaling and triggers the release of IL-6 and IL-8 from gingival and endothelial cells [53]. These events transform the periodontal vascularization into a gateway for the spillover of inflammatory mediators. Once in the bloodstream, these cytokines act as molecular messengers that can reprogram immune activity in distant tissues [54]. This process is bidirectional: systemic inflammatory conditions such as diabetes and metabolic syndrome amplify local periodontal inflammation through the same mediators, establishing what has recently been termed an “inflammatory connectome”—a dynamic communication network linking oral and systemic inflammation [7,55]. The vascular endothelium is both a target and an amplifier of this network.

Circulating IL-1β and TNF-α induce expression of adhesion molecules ICAM-1 (intercellular adhesion molecule 1), VCAM-1 (vascular cell adhesion molecule 1), E-selectin, promoting leukocyte recruitment and vascular permeability [56]. In parallel, bacterial components, such as *Porphyromonas gingivalis* lipopolysaccharide, outer membrane vesicles, and microbial DNA (deoxyribonucleic acid), may enter the bloodstream through inflamed capillaries [57]. Pathogens can survive intracellularly within monocytes and dendritic cells, disseminating via a “Trojan horse” mechanism to distant organs such as the liver or placenta, perpetuating systemic cytokine release [58]. These microbial and host-derived mediators stimulate NF-κB and JAK–STAT (Janus kinase—signal transducer and activator of transcription) pathways in monocytes, macrophages, and endothelial cells, promoting hepatic synthesis of acute-phase reactants such as CRP and serum amyloid A [59]. The resulting chronic cytokinemia mirrors the systemic inflammatory response syndrome, characterized by persistent complement activation, vascular leakage, and metabolic stress [60,61]. Although periodontitis does not trigger fulminant sepsis, the low-grade systemic inflammation it induces drives continuous endothelial stress and immune activation—hallmarks of systemic immune reprogramming. It is worth mentioning that these circulating mediators can cross biological barriers. Pro-inflammatory molecules and microbial fragments can break the blood–brain barrier, as demonstrated experimentally [3]. These findings emphasize the role of the vascular system as a channel for inflammatory communication between the oral and systemic environments.

#### 2.1.3. Consequence: Chronic Activation of Myeloid and Endothelial CELLS → “Immune Fatigue”

The sustained stimulation of myeloid and endothelial cells by circulating IL-6, TNF-α, and IL-1β ultimately induces immune fatigue—a maladaptive, exhaustion-like state of myeloid and endothelial cells (hereafter referred to as ‘immune fatigue’) [62]. Continuous NF-κB and STAT3 (signal transducer and activator of transcription 3) signaling leads to oxidative stress, mitochondrial dysfunction, and diminished responsiveness to novel antigens [63]. As a result, phagocytic efficiency, cytokine modulation, and endothelial repair are progressively impaired. Comparable mechanisms are recognized in other chronic inflammatory settings. Prolonged activation of innate immune cells in the brain during HIV (human immunodeficiency virus) infection or chronic opioid exposure causes epigenetically mediated immune fatigue, characterized by loss of microglial homeostasis [64]. Likewise, metabolic stress-induced immune fatigue has been identified as a barrier to the efficacy of CAR T-cell (chimeric antigen receptor T cell) therapy in glioblastoma, where chronic inflammation suppresses cytotoxic function [65]. Analogously, in periodontitis, persistent activation of circulating monocytes and endothelial cells results in a paradoxical immune landscape that is hyper-inflammatory yet functionally depleted [66]. This “trained-yet-tired” phenotype exemplifies the systemic immune reprogramming driven by chronic oral inflammation, undermining both tissue homeostasis and host antitumor immunity [67]. This paradoxical state forms the biological bridge to the next phase of systemic immune adaptation—epigenetic and metabolic reprogramming of myeloid cells—addressed in Section 2.2. (Trained Immunity and Myeloid Reprogramming). These processes define the systemic inflammatory axis of periodontitis and establish the mechanistic basis for the epigenetic and metabolic reprogramming of innate immune cells discussed in the following section.

### 2.2. Trained Immunity and Myeloid Reprogramming

These processes establish the systemic inflammation observed in periodontitis. They also lay the foundation for how innate immune cells are changed at the epigenetic and metabolic levels, which will be discussed in the next section.

Chronic periodontitis induces sustained alterations in the innate immune system via trained immunity [68]. During this process, monocytes and macrophages undergo epigenetic and metabolic modifications [69]. In contrast to adaptive immune memory, trained immunity is an innate, memory-like response that enables cells to mount responses to subsequent stimuli [70]. In the context of periodontitis, this mechanism contributes to ongoing inflammation and immune dysregulation, even following local therapeutic interventions [71].

This reprogramming is evident as periodontitis induces both epigenetic and metabolic reprogramming of monocytes/macrophages [72,73].

#### 2.2.1. Periodontitis Induces Epigenetic and Metabolic Reprogramming of Monocytes/Macrophages

Periodontitis-associated bacteria, *Porphyromonas gingivalis* and *Fusobacterium nucleatum*, cause extensive transcriptional and metabolic reprogramming in circulating monocytes. Noz et al. (2021) found that exposure to periodontal pathogens creates a trained phenotype. This phenotype is characterized by increased cytokine release and accelerated atherogenesis [66]. Four years later, Zhang et al. (2025) reported pronounced metabolic rewiring in macrophages from periodontitis. These cells shift toward aerobic glycolysis and change their mitochondria. As a result, they stay in a persistent pro-inflammatory state [74].

Trained macrophages exhibit lasting epigenetic and metabolic changes that reinforce one another. Daskalaki et al. (2025) found that innate immune training is regulated by histone methylation (H3K4me3) and histone acetylation (H3K27ac) as the promoters of inflammatory genes. Increased glycolysis and glutaminolysis support constant cytokine production [72]. Ferreira et al. (2024) described how TCA (tricarboxylic acid) cycle and mevalonate pathway intermediates help remodel chromatin and keep myeloid cells activated [75].

Mitochondrial homeostasis also shapes this process. Cheng et al. (2025) found that vitamin A derivatives restore mitochondrial function through the JAK–STAT pathway. This action counters hyperinflammatory reprogramming in macrophages linked to periodontitis [76], persistent periodontitis induces genetic polymorphism of interleukins [77].

All these studies show that trained immunity stems from an integrated epigenetic and metabolic network. This network controls macrophage persistence and shapes systemic inflammatory responses.

#### 2.2.2. Mechanism: TLR Signaling, Histone Modification, and Glycolytic Shift

At the mechanistic level, the induction of trained immunity begins with pattern recognition receptor (PRR) activation, primarily through Toll-like receptors (TLR2/TLR4) and NOD2, which sense periodontal bacterial components such as LPS and peptidoglycans. Hajishengallis (2021) elucidated that these receptors trigger NF-κB and MAPK cascades that open chromatin at inflammatory loci, facilitating transcriptional priming of myeloid cells [4]. Lauterbach et al. (2019) revealed that TLR engagement activates ATP-citrate lyase (ACL), which converts citrate into acetyl-CoA for histone acetylation, creating a permissive chromatin landscape that sustains inflammatory gene expression [78]. Similarly, Irizarry-Caro et al. (2020) demonstrated that the TLR adapter BCAP regulates macrophage transition from inflammatory to reparatory states by modulating histone acetylation dynamics [79]. A groundbreaking layer of this regulation is histone lactylation, linking cellular metabolism directly to chromatin activity. Xie et al. (2022) and Wang et al. (2023) showed that lactate—a glycolytic end-product—serves as a substrate for histone lysine lactylation, activating genes that balance inflammation and tissue repair [80,81]. This phenomenon, induced by TLR-driven glycolytic flux, provides an elegant explanation for how metabolic shifts perpetuate inflammatory memory. Namgaladze et al. (2023) identified early glycolytic activation as a key mediator coupling innate immune signaling to histone modification, while Li et al. (2024) demonstrated that lactylation and glycolysis act synergistically to regulate macrophage function in both infection and cancer [82,83]. Wu et al. (2023) expanded these insights, showing that epigenetic marks—acetylation, methylation, lactylation—govern macrophage polarization and cytokine production during sepsis-like inflammation [84]. More recently, Bao et al. (2025) and Peng et al. (2025) revealed that reactive oxygen species (mROS)-induced glycolytic shifts promote histone lactylation at H3K18 and H3K23 residues, stabilizing pro-inflammatory transcriptional programs [85,86]. Yao et al. (2024) placed lactylation among nine novel post-translational histone modifications—alongside crotonylation and succinylation—integrating it into the broader regulatory network of inflammation and immunity [87]. These findings converge on a unified model: TLR activation triggers metabolic reprogramming toward glycolysis, generating lactate that fuels histone lactylation, which in turn locks macrophages into a “trained” transcriptional state. The result is sustained low-grade inflammation even after pathogen clearance—a hallmark of systemic immune reprogramming in periodontitis.

#### 2.2.3. Systemic Effects: Persistent Hyperresponsiveness → Impaired Immunoregulation

The downstream consequence of this reprogramming is a systemic state of persistent hyperresponsiveness accompanied by immunoregulatory dysfunction. Trained monocytes maintain high basal cytokine output, driving chronic low-grade inflammation and oxidative stress. Breivik et al. (2025) demonstrated that persistent hyperactivation of the neuroimmune stress axis dysregulates TNF-α responsiveness, linking inflammation, stress hormones, and immune fatigue [88]. Simultaneously, mast cells act as amplifiers of this systemic state. Tsai et al. (2013) showed that mast cells function not only as effector cells but also as antigen-presenting cells, influencing adaptive immunity and perpetuating cytokine cascades during chronic inflammation [89]. Chronic exposure to IL-6 and TNF-α also induces glucocorticoid resistance in immune cells, blunting the anti-inflammatory effects of cortisol and prolonging immune activation [90]. According to Xu et al. (2025), corticosterone exerts dual effects—initially suppressing inflammation but ultimately causing maladaptive immunosuppression through feedback dysregulation [91]. The net result is an oscillation between hyperactivation and immune exhaustion, characteristic of systemic immune reprogramming. Comparable dynamics are observed in other chronic inflammatory diseases. In asthma, Chapman et al. (2015) described persistent airway hyperresponsiveness as a form of cellular memory resulting from chronic cytokine exposure [92], while Reza et al. (2025) confirmed that eosinophil-driven oxidative stress maintains a similar hyperreactive–exhausted cycle [93]. Abbas et al. (2025) further demonstrated that persistent hyperglycemia in diabetes mellitus suppresses immune response while maintaining systemic inflammation, a pattern similar to that observed in periodontitis [94]. This dysregulation extends to the hematopoietic niche, where chronic cytokinemia promotes myelopoietic bias at the expense of lymphopoiesis. The bone marrow thus becomes a reservoir of pre-activated monocytes and neutrophils, perpetuating inflammation even in the absence of microbial triggers. Kleniewska et al. (2024) linked this phenomenon to oxidative stress and dysbiosis, while Hirobumi et al. (2025) demonstrated that chronic mucosal inflammation disrupts neuroimmune coordination, further impairing systemic immunoregulation [95,96].

In summary, chronic periodontal inflammation has been shown to induce persistent innate immune alterations consistent with trained immunity–like reprogramming, although direct causal clinical validation remains limited. Through TLR signaling, histone modification, and metabolic adaptation, monocytes and macrophages acquire a trained yet dysfunctional phenotype—hyperresponsive but regulatory-deficient. This systemic immune reprogramming contributes to persistent inflammation, endothelial dysfunction, and reduced immune flexibility, representing a key biological bridge between periodontitis, metabolic disorders, and altered responses to cancer immunotherapy. These interconnected mechanisms are summarized in Table 1, providing an integrated overview of systemic immune reprogramming driven by chronic periodontitis.

This maladaptive immune conditioning extends to the adaptive compartment, where T-cell dysregulation further perpetuates systemic immune reprogramming, as explored in Section 2.3.

### 2.3. Adaptive Immunity and T-Cell Dysregulation

Building upon the innate immune alterations described previously, chronic periodontitis profoundly alters adaptive immune homeostasis, driving a sustained imbalance between effector and regulatory T-cell subsets and promoting features of immune exhaustion that mirror those observed in chronic infections and cancer. The continuous antigenic and inflammatory stimulation generated by the subgingival biofilm reshapes the T-cell landscape through cytokine-driven differentiation, checkpoint activation, and loss of cytotoxic competence. These adaptive immune perturbations extend systemically, influencing inflammatory homeostasis and potentially modulating responses to immunotherapy.

#### 2.3.1. Skewing Toward Th17 and Treg Imbalance

The Th17/Treg axis represents a central regulatory node in the immunopathogenesis of periodontitis. Th17 cells, characterized by IL-17A, IL-22, and RORγt expression, promote neutrophil recruitment and osteoclastic bone resorption, whereas regulatory T cells (Tregs), expressing FOXP3 and secreting IL-10 and TGF-β, exert immunosuppressive and tissue-protective effects. A delicate equilibrium between these subsets is essential for periodontal homeostasis; disruption of this balance leads to persistent inflammation and progressive tissue destruction. Multiple studies have demonstrated that individuals with chronic periodontitis exhibit an elevated Th17/Treg ratio both in gingival tissues and peripheral blood [91,97,98,99]. Gao et al. (2017) first documented that increased Th17 frequency and reduced Treg activity correlate with disease severity and alveolar bone loss [97]. Subsequent analyses by Wang et al. (2021) confirmed that gingivitis and periodontitis are accompanied by elevated IL-17A, IL-6, and IL-23 levels, promoting Th17 polarization and inhibiting Treg differentiation [98]. The functional interplay between dendritic cells and CD4^+^ T cells further amplifies this disequilibrium: Porphyromonas gingivalis LPS activates CD40 on dendritic cells, increasing IL-6 and IL-23 production and enhancing STAT3-mediated Th17 differentiation [91]. Conversely, Treg depletion or dysfunction accelerates periodontal breakdown. Deng et al. (2022) emphasized that Tregs mitigate osteoclastic activity via RANKL/OPG modulation, while their decline promotes bone loss [99]. Aging and T-cell senescence exacerbate this imbalance, as shown by González-Osuna et al. (2022, MDPI), who reported that senescent CD4^+^CD28^−^ lymphocytes exhibit reduced Treg functionality and enhanced Th17 cytokine expression [100]. Therapeutic modulation of this axis remains feasible: all-trans retinoic acid (ATRA) and traditional formulations such as Yunnan Baiyao have been shown to restore Th17/Treg equilibrium and reduce periodontal inflammation in experimental models [101]. Collectively, these findings indicate that Th17/Treg imbalance represents not merely a byproduct of inflammation but a driver of chronic immune activation in periodontitis.

#### 2.3.2. Chronic Antigen Exposure Leads to T-Cell Exhaustion and PD-1 Upregulation

Sustained exposure to bacterial antigens and inflammatory mediators in chronic periodontitis results in functional exhaustion of T cells, a process analogous to that observed in chronic viral infections and malignancies, characterized by reduced IL-2, IFN-γ, and granzyme B expression, alongside decreased cytotoxic granule release that collectively limit their effector competence [102]. This phenomenon involves progressive loss of proliferative capacity, reduced cytokine secretion, and increased expression of inhibitory receptors, most notably programmed cell death-1 (PD-1) and its ligand PD-L1. Jubel et al. (2020) demonstrated that continuous antigenic stimulation leads to persistent PD-1 expression on both CD4^+^ and CD8^+^ T cells, impairing their ability to mediate pathogen clearance [103]. In the context of periodontitis, Bailly (2020) proposed that activation of the PD-1/PD-L1 checkpoint contributes to local immunosuppression and disease chronicity [35]. Recent work by Wang et al. (2024) confirmed this hypothesis by demonstrating increased PD-1^+^CD8^+^ T cells and PD-L1^+^ macrophages in periodontal tissues, concomitant with reduced IFN-γ^+^ effector T-cell populations [37]. The PD-1/PD-L1 axis mediates immune tolerance by dampening TCR signaling, reducing cytotoxic granule release, and promoting Treg expansion. Chronic microbial exposure, including to *Porphyromonas gingivalis* outer membrane vesicles, maintains persistent low-grade PD-1 induction, contributing to local and systemic immune exhaustion [104]. Comparable mechanisms have been described in other chronic infections such as CMV and HCV, where prolonged antigen stimulation induces PD-1, TIM-3, and LAG-3 expression, leading to anergy and loss of effector function [103]. Importantly, similar immunophenotypes have been detected in apical and gingival lesions, suggesting that oral inflammation can shape systemic T-cell profiles [105]. Furthermore, epigenetic stability of PD-1 expression—mediated through histone modifications and DNA methylation—locks exhausted T cells into a hyporesponsive state, limiting their capacity for reinvigoration even upon checkpoint blockade [106]. Thus, chronic antigen exposure in the periodontium may precondition the immune system toward exhaustion, undermining the host’s ability to control both microbial infections and neoplastic cells.

#### 2.3.3. Impaired Cytotoxic Function May Blunt Immunotherapy Efficacy

An emerging body of evidence indicates that periodontitis-induced immune exhaustion extends its influence to systemic immune competence, potentially modulating responses to immunotherapy in oncology [71]. Chronic periodontal inflammation induces not only CD8^+^ T-cell exhaustion but also alters natural killer (NK) cell cytotoxicity, macrophage polarization, and antigen-presenting cell function [107]. These systemic effects may plausibly influence pathways relevant to immune checkpoint inhibitor responsiveness, although direct clinical validation remains scarce. Pai et al. (2023) reviewed the impact of periodontal disease on cancer immunotherapy outcomes, highlighting that oral dysbiosis and the resulting chronic inflammatory milieu can impair immune responsiveness to PD-1/PD-L1 blockade [36]. Indeed, patients with untreated periodontitis exhibit systemic elevations in IL-6 and TNF-α, cytokines known to promote myeloid-derived suppressor cell (MDSC) expansion and T-cell suppression, thereby reducing checkpoint inhibitor efficacy [108]. Conversely, some preliminary observations suggested that active periodontal treatment may enhance the effectiveness of immunotherapy by lowering systemic inflammation and restoring immune homeostasis [109].

At the mechanistic level, the immunosuppressive tumor microenvironment (TME) shares multiple features with chronic periodontal inflammation. Kzhyshkowska et al. (2024) noted that tumor-associated macrophages (TAMs) execute similar immunoregulatory functions as periodontal macrophages—producing IL-10, TGF-β, and VEGF, which collectively suppress cytotoxic activity and promote immune escape [110]. Moreover, chronic IL-6 signaling from the periodontium can activate STAT3 in circulating lymphocytes, further attenuating cytotoxic function and impairing responses to checkpoint blockade [111]. Additional evidence supports a role for co-inhibitory receptor synergy: TIM-3 and LAG-3 co-expression with PD-1 on CD8^+^ T cells correlate with markedly reduced cytotoxicity and diminished IFN-γ production [112]. The result is a systemic immunological landscape characterized by “double exhaustion”, in which both adaptive and innate cytotoxic arms are suppressed. This state may explain the lower responsiveness to immunotherapy observed in patients with coexisting chronic inflammatory diseases, including periodontitis. Finally, experimental and clinical data converge to suggest that immune reactivation therapies could benefit from concurrent management of oral inflammation. As emphasized by Zhang et al. (2025), microbial and inflammatory products derived from the oral cavity may modulate tumor-associated microbiota and immune signaling, ultimately influencing therapeutic efficacy [113]. Periodontal treatment, through modulation of systemic cytokine levels and reduction in immune checkpoint activation, may represent a conceptually promising adjunct strategy to potentiate antitumor immunity, but requires clinical validation. This bidirectional interplay between periodontal and systemic immunity represents a critical yet underexplored determinant of cancer immunotherapy responsiveness, warranting further translational investigation [113]. In summary, chronic periodontitis drives maladaptive T-cell reprogramming characterized by Th17/Treg disequilibrium, PD-1–mediated exhaustion, and suppression of cytotoxic function. These alterations propagate systemically, creating an immunological environment of chronic activation yet impaired effector potential—a hallmark of immune exhaustion. The convergence between periodontal and tumor immunopathology underscores the importance of periodontal health not only for local tissue integrity but also for optimizing systemic immune responsiveness and improving outcomes in the era of precision immunotherapy. Together, these innate and adaptive immune alterations outline a multilevel reprogramming of the host immune system in periodontitis. The convergence of cytokine spillover, myeloid training, and T-cell exhaustion creates a persistent state of systemic inflammation and immune dysregulation.

Collectively, these epigenetic and metabolic alterations establish a durable state of trained immunity that links chronic periodontal inflammation to sustained systemic immune activation.

## 3. Shared Molecular Pathways Between Periodontitis and Tumor Immunity

Multiple studies have shown that periodontitis and tumor-associated immune dysfunction share molecular pathways that regulate inflammation, immune activation, and suppression (Table 2). NF-κB activation has been implicated in inflammatory circuits that can support tumor-promoting immune modulation, although direct causal links in the context of periodontitis remain to be confirmed. It drives the transcription of cytokines IL-6, IL-8, and TNF-α. These cytokines maintain chronic inflammation and induce pathological tissue remodeling. STAT3 activation by these cytokines further supports the pathway. STAT3 links chronic inflammation to compromised antigen presentation. It also drives the expansion of immunosuppressive cell populations and suppresses adaptive immune responses. STAT3 represents one of the pathways through which chronic periodontal inflammation could intersect with oncogenic immune regulation, but further evidence is required to establish causality. These findings reveal a link between the diseases through shared signaling cascades that drive immune dysfunction.

Dysregulation of the PD-1/PD-L1 immune checkpoint axis is another major point of convergence. Periodontitis-associated inflammatory signaling may promote PD-L1 upregulation in immune and stromal compartments, potentially contributing to an immunosuppressive milieu relevant to checkpoint blockade responsiveness. Increased PD-L1 expression is observed in both periodontal lesions and many cancerous tissues. Here, it contributes to T-cell exhaustion and reduced cytotoxic activity. Key periodontal pathogens, especially *Porphyromonas gingivalis*, influence these pathways. *Porphyromonas gingivalis* induces PD-L1 and IL-10 expression in dendritic cells and macrophages. This action promotes a tolerogenic myeloid phenotype, resembling tumor-mediated immune evasion mechanisms.

**Figure 2:**
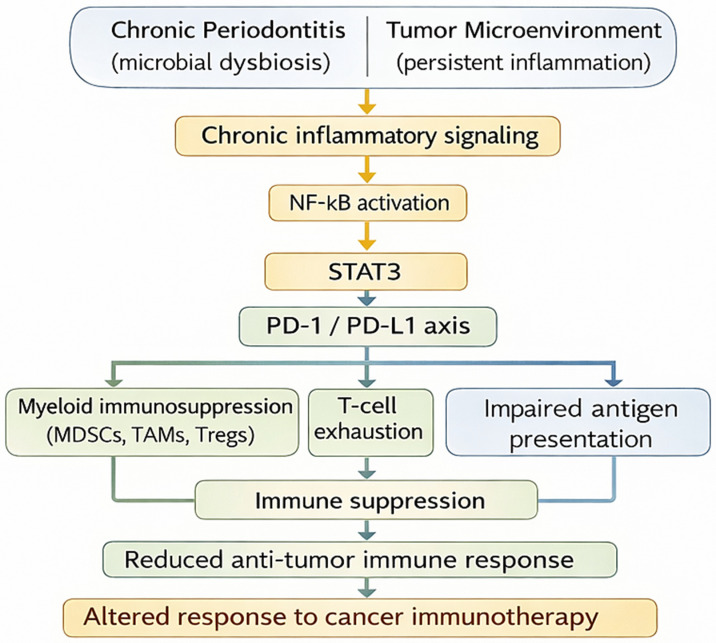
Shared immune regulatory axis in chronic inflammation and cancer. Legend: Chronic periodontal inflammation and the tumor microenvironment engage a common immune regulatory axis centered on NF-κB, STAT3, and PD-1/PD-L1 signaling. Distinct inflammatory triggers—microbial dysbiosis in periodontitis and persistent inflammatory cues in cancer—converge on this shared regulatory logic, promoting immune checkpoint activation, myeloid immunosuppression, and T-cell exhaustion. This convergence illustrates how chronic oral inflammation may precondition systemic immune responses in ways that influence tumor immune evasion and responsiveness to cancer immunotherapy.

Overall, the evidence summarized in Table 2 suggests that periodontitis and oral dysbiosis may influence systemic immune regulation through mechanisms relevant to tumor immune escape and immunotherapy response (Figure 2). Preclinical and emerging clinical data indicate that periodontal inflammation is associated with immune checkpoint activation, immunosuppressive cell expansion, and altered treatment outcomes. Perturbations of the oral microbiome may further contribute to this process by promoting sustained local inflammation and facilitating the dissemination of inflammatory mediators and microbial products into the systemic circulation [7,121,122]. These findings support the concept that periodontal disease may represent a modifiable host factor affecting antitumor immunity and warrants further prospective investigation in immunotherapy-treated patients.

Beyond inflammatory cytokine-driven immune reprogramming, new molecular regulatory pathways in oral malignancies may also influence immunotherapy responsiveness. For example, cuproptosis—a recently described copper-dependent form of programmed cell death—has been implicated in tumor progression and immune modulation. Recent work identified a cuproptosis-related long non-coding RNA (lncRNA) signature that predicts prognosis and therapeutic response to immune checkpoint blockade in OSCC. Such findings highlight that immune responsiveness in oral cancers reflects a convergence of inflammatory, metabolic, and epigenetic regulatory networks, supporting the relevance of immune conditioning within the oral niche for cancer immunotherapy outcomes [123].

Nowadays, the clinical efficacy of immune checkpoint-targeting monoclonal antibodies (mAbs), including anti–PD-1, anti–PD-L1, and anti–CTLA-4 therapies, is increasingly recognized to depend not only on tumor-intrinsic features but also on extrinsic host factors such as infection status, environmental exposures, lifestyle, and systemic inflammatory burden. A recent review emphasized that these variables can significantly shape immune responsiveness and contribute to therapeutic benefit and immune-related adverse events (irAEs) [124]. Such observations highlight the need for predictive biomarkers and modifiable host factors to optimize immunotherapy outcomes. In this context, chronic inflammatory diseases such as periodontitis may represent an underappreciated source of systemic immune perturbation with potential implications for mAb efficacy and toxicity.

Taken together, these shared molecular mechanisms suggest that chronic oral inflammation may precondition the systemic immune milieu toward a more suppressive or tolerance-skewed state, with possible consequences for antitumor immunity and responsiveness to immune checkpoint blockade. This convergence underscores the importance of considering periodontal immune perturbations alongside other host factors that influence cancer immunotherapy outcomes.

From a translational perspective, convergence of periodontal and tumor-associated immune regulatory pathways may contribute to interindividual variability in immunotherapy outcomes. Baseline systemic inflammation, elevated IL-6 or CRP levels, increased myeloid-derived suppressor cell frequencies, and heightened PD-1 expression on circulating T cells have all been associated with reduced responsiveness to immune checkpoint inhibitors. Periodontitis may represent a systemic inflammatory condition capable of influencing immune pathways relevant to checkpoint inhibitor responsiveness, although direct clinical evidence remains scarce. Periodontitis-associated immune reprogramming may therefore represent a clinically relevant modifier of immune fitness, with potential implications for patient stratification and therapeutic optimization in immuno-oncology.

Evidence indicates that periodontal therapy may reduce systemic inflammatory burden, supporting the potential for partial immune rebalancing following oral inflammatory control. Meta-analyses demonstrate that non-surgical periodontal therapy is associated with reductions in circulating inflammatory markers, such as IL-6 (moderate certainty) and CRP (lower certainty), although heterogeneity in populations, protocols, and follow-up durations limits the strength of these conclusions [125,126]. Similarly, interventional studies have reported decreases in serum IL-6 and acute-phase reactants after periodontal treatment in both systemically healthy individuals and those with varying disease severities [46,127]. However, the immune parameters assessed are variable and often restricted to soluble biomarkers. Notably, prospective trials evaluating whether periodontal intervention influences immune checkpoint inhibitor efficacy or immune-related toxicity in cancer patients are currently lacking. As such, periodontal therapy should be regarded as a biologically plausible yet clinically unvalidated immunomodulatory approach, necessitating dedicated immuno-oncology studies to determine its significance for cancer immunotherapy outcomes.

## 4. Confounding Factors and Modifiers of the Periodontitis–Cancer Immunity Axis

Interpretation of links between periodontitis, systemic immune reprogramming, and cancer outcomes must consider several important confounding and modifying variables. Smoking is a major shared risk factor that influences periodontal tissue destruction, systemic inflammation, and immune suppression, while also shaping cancer susceptibility and response to immunotherapy [128]. Likewise, metabolic diseases such as diabetes and obesity are strongly associated with chronic low-grade inflammation, altered myeloid function, and impaired immune surveillance, potentially amplifying both periodontal and tumor-promoting immune phenotypes [4,129]. In addition, inter-individual variability in the oral and gut microbiome may contribute to differences in immune training, cytokine profiles, and checkpoint responsiveness [130]. Socioeconomic status and healthcare access further modulate periodontal burden, comorbidity prevalence, and cancer treatment disparities. These overlapping determinants underscore that periodontitis may act as one component within a broader inflammatory and environmental network, and future studies must incorporate careful adjustment for these confounders when evaluating causal relationships and immunotherapy outcomes.

These confounders highlight the necessity of well-controlled prospective studies adjusting for behavioral, metabolic, microbial, and socioeconomic determinants [131].

## 5. Knowledge Gaps, Limitations and Future Research Directions

Despite increasing recognition of the systemic immunological consequences of periodontitis, critical knowledge gaps remain regarding its role in shaping antitumor immunity and responses to cancer immunotherapy. Much of the current evidence is derived from preclinical models, in vitro experiments, cross-sectional human studies, or retrospective clinical observations. Although these data consistently support associations between periodontal inflammation, systemic immune dysregulation, and oncologic outcomes, prospective investigations designed to establish causality and temporal relationships are still lacking. In particular, direct clinical studies examining periodontal disease as a determinant of immune checkpoint blockade responsiveness remain scarce. Future research should incorporate rigorous periodontal phenotyping, systemic immune profiling, and immunotherapy outcome assessment in well-controlled longitudinal cohorts, and evaluate whether periodontal treatment may improve immune fitness and therapeutic response.

A major unresolved question concerns the extent to which periodontal inflammation independently contributes to systemic immune reprogramming in the context of complex comorbid states. Periodontitis frequently coexists with metabolic disorders, cardiovascular disease, smoking-related pathology, and aging-associated immune alterations, all of which engage overlapping inflammatory and immunosuppressive pathways. Disentangling the specific contribution of oral inflammatory burden from these confounding factors remains a key challenge for future clinical and translational research.

Another important gap relates to heterogeneity in disease characterization. Periodontitis encompasses a spectrum of clinical phenotypes, microbial compositions, and inflammatory intensities, yet most immunological studies do not stratify patients by disease severity, duration, or microbial signatures. Given that trained immunity and immune exhaustion are cumulative and context dependent, refined phenotyping will be essential to clarify how different periodontal states differentially influence systemic immune tone.

From a translational perspective, the impact of periodontal immune perturbations on cancer immunotherapy outcomes remains largely unexplored in prospective clinical settings. Future studies should incorporate standardized periodontal assessments into immunotherapy trials and longitudinal cancer cohorts, alongside systemic inflammatory markers, immune phenotyping, and treatment response metrics. Interventional studies evaluating whether periodontal therapy can recalibrate systemic immune parameters or enhance responsiveness to immune checkpoint blockade would be particularly informative.

Finally, mechanistic insights into how periodontal inflammation imprints durable immune memory warrant further investigation. Integrated multi-omics approaches—including epigenomic, metabolomic, and microbiome profiling—will be critical to elucidate how oral inflammatory signals shape long-term immune programming and interact with tumor-associated immune regulation. Addressing these knowledge gaps will be essential for translating oral–systemic immunology into actionable strategies within precision immunotherapy frameworks.

The proposed overlap between periodontal inflammation and immune checkpoint regulation is supported by shared inflammatory mediators, preclinical models, and indirect clinical associations. Although these convergent pathways offer a compelling mechanistic framework, definitive causal evidence linking periodontitis-driven NF-κB/STAT3 activation to altered immunotherapy response in cancer patients is currently limited. Prospective studies that incorporate periodontal status, systemic immune profiling, and immunotherapy outcomes are necessary to validate these interactions.

## 6. Clinical Implications and Evidence Boundaries

A clear distinction should be maintained between clinically established observations and emerging mechanistic hypotheses. Periodontitis is consistently associated with increased systemic inflammatory burden and has been linked epidemiologically to elevated cancer risk and poorer oncologic outcomes. However, most proposed associations with immune checkpoint regulation, trained immunity, and therapeutic response are primarily supported by preclinical models, indirect immune profiling, or associative human studies. While periodontal inflammation is a biologically plausible modifier of systemic immune fitness, its role as a clinically actionable determinant of immune checkpoint inhibitor efficacy remains unvalidated. Prospective clinical trials are necessary before periodontal assessment or intervention can be formally incorporated into immunotherapy decision-making.

From a translational perspective, integrating oral health assessment into immunotherapy management may be accomplished through practical, low-burden strategies. For instance, baseline periodontal screening and history-taking could be included in pre-treatment evaluations prior to the initiation of immune checkpoint blockade, particularly for patients with established risk factors such as smoking or diabetes. Patients with moderate-to-severe periodontal disease may be referred for dental or periodontal consultation as part of multidisciplinary supportive care. Standardized oral hygiene interventions and periodontal therapy, when clinically indicated, could help reduce systemic inflammatory burden, although their effects on immunotherapy efficacy or immune-related adverse events remain unproven. Collaboration among oncologists, immunologists, and dental specialists will be necessary to determine whether periodontal assessment should ultimately be integrated into immuno-oncology clinical pathways.

## 7. Conclusions

In summary, chronic periodontitis emerges as a systemic immunoinflammatory condition capable of reshaping immune responsiveness beyond the oral cavity. Through sustained cytokine signaling, trained immunity of myeloid cells, and adaptive immune exhaustion, periodontal inflammation may lower the immune set point required for effective antitumor immunity. Recognizing periodontal immune status as a modifiable host factor could refine patient stratification and open new avenues for immune recalibration in cancer immunotherapy.

## Figures and Tables

**Table 1:** Systemic immunological alterations associated with periodontitis.

Mechanistic Axis	Primary Mediators/Pathways	Cellular Targets	Functional Consequences
Cytokine spillover and chronic inflammation	IL-1β, IL-6, TNF-α, CRP; NF-κB, JAK–STAT	Monocytes, endothelial cells, hepatocytes	Systemic low-grade inflammation; endothelial activation; acute-phase response
Dissemination of inflammatory mediators	PGE_2_, LPS, bacterial DNA, outer-membrane vesicles; TLR2/TLR4	Leukocytes, endothelial cells, dendritic cells	Entry of inflammatory signals into bloodstream; immune reprogramming at distant sites
Myeloid and endothelial exhaustion (“immune fatigue”)	Persistent IL-6/TNF-α/IL-1β signaling; STAT3 hyperactivation	Monocytes/macrophages, endothelial cells	Mitochondrial dysfunction; impaired phagocytosis; oxidative stress
Epigenetic and metabolic reprogramming (trained immunity)	H3K4me3, H3K27ac; glycolysis, mevalonate, TCA intermediates	Monocytes, macrophages	Persistent pro-inflammatory “memory”; sustained cytokine output
Histone lactylation and metabolic signaling	Lactate, ROS; glycolytic flux; TLR2/TLR4	Macrophages, myeloid progenitors	Lactate-dependent chromatin remodeling; stabilized inflammatory transcription
Neuroendocrine–immune dysregulation	TNF-α, IL-6, cortisol/corticosterone	Mast cells, myeloid cells, HPA axis	Glucocorticoid resistance; feedback dysregulation
Th17/Treg imbalance	IL-17A, IL-23, IL-6, TGF-β; FOXP3	CD4^+^ T cells, dendritic cells	Excess Th17 activation; reduced Treg function
T-cell exhaustion and checkpoint upregulation	PD-1/PD-L1, TIM-3, LAG-3	CD4^+^/CD8^+^ T cells, macrophages	Hyporesponsiveness; decreased effector cytokines
Cytotoxic impairment and immunotherapy interference	IL-6/TNF-α→STAT3; MDSCs; TAMs	CD8^+^ T cells, NK cells, APCs	Suppressed cytotoxic activity; decreased IFN-γ

Legend: Summary of key systemic immune alterations linked to chronic periodontitis, including major cytokines, immune cell reprogramming pathways, and resulting systemic effects. The table highlights the interactions between innate and adaptive immunity that contribute to systemic immune reprogramming and potential modulation of cancer immunotherapy response. Abbreviations: IL—interleukin; TNF-α—tumor necrosis factor alpha; CRP—C-reactive protein; NF-κB—nuclear factor kappa-B; JAK—Janus kinase; STAT3—signal transducer and activator of transcription 3; TLR—Toll-like receptor; NOD2—nucleotide-binding oligomerization domain-containing protein 2; mROS—mitochondrial reactive oxygen species; ACL—ATP-citrate lyase; ICAM-1—intercellular adhesion molecule-1; VCAM-1—vascular cell adhesion molecule-1; HPA—hypothalamic–pituitary–adrenal; Th17—T helper 17; Treg—regulatory T cell; PD-1—programmed cell death-1; PD-L1—programmed death ligand 1; MDSC—myeloid-derived suppressor cell; TAM—tumor-associated macrophage; FOXP3—fork-head box P3.

**Table 2:** Evidence linking periodontitis, immune dysregulation, and cancer immunotherapy outcomes.

Tumor Type	Key Finding	Relevance	Reference
Oral squamous cell carcinoma (OSCC)	Periodontitis promoted OSCC development and was associated with sustained alterations in the oral microbiota and tumor immune microenvironment. Periodontitis-derived oral microbiota, dominated by *Porphyromonas*, persisted throughout tumor progression and directly activated IL-17^+^ γδ T cells, triggering IL-17/STAT3 signaling and promoting M2 tumor-associated macrophage infiltration.	Demonstrates that the oral microbiota associated with periodontitis can shape a pro-tumorigenic immune microenvironment through IL-17–STAT3–TAM pathways.	Wei et al., 2022 [114]
Cancer (general)	Activation of the PD-1/PD-L1 immune checkpoint pathway in periodontal tissues impairs T-cell function and contributes to chronic tissue inflammation and destruction.	Chronic periodontal PD-1/PD-L1 activation may promote systemic T-cell exhaustion and facilitate tumor immune escape mechanisms.	Liu et al., 2023 [115]
Oral cancer; (animal models)	*Porphyromonas gingivalis* infection upregulates PD-L1 expression on dendritic cells via Akt–STAT3 signaling, suppresses CD8^+^ T-cell cytotoxicity, and accelerates oral tumor growth.	Targeting *Porphyromonas gingivalis*-driven immune checkpoint activation or its downstream signaling pathways may represent a therapeutic strategy to enhance the efficacy of immune checkpoint blockade.	Ren et al., 2023 [116]
Squamous cell carcinoma; colorectal cancer	Overlap observed between immune-related adverse events (irAEs) of immune checkpoint inhibitors and comorbid conditions frequently associated with periodontitis.	Proposes that periodontal disease and oral dysbiosis may influence immunotherapy response	Pai Si et al., [36]
Oral cancer; prostate cancer (animal models)	Experimental periodontitis enhanced tumor growth, increased Treg cells, M2-polarized tumor-associated macrophages, and PD-1^+^ CD8^+^ T cells, while suppressing cytotoxic T-cell activity.	Provides experimental evidence supporting the role of periodontitis in promoting tumor growth and immune suppression via checkpoint-related pathways in vivo.	Wang S. et al., 2024 [37]
Cancer (general)	A bidirectional relationship exists between increased PD-L1 expression, periodontitis, and epithelial–mesenchymal transition. Periodontopathogenic bacteria and their components upregulate PD-L1, while exosomal PD-L1 interacts with PD-1 on T cells, inhibiting T-cell activation and proliferation.	Identifies PD-L1 as a mechanistic interlink between chronic periodontal inflammation and cancer, whereby immune checkpoint-mediated immunosuppression and sustained inflammation synergistically support tumor immune escape and cancer progression.	Groeger et al., 2024 [117]
Cancer (mixed cohorts)	Patients treated with immune checkpoint inhibitors showed approximately a two-fold higher risk of newly diagnosed periodontitis. Immune-related periodontitis as an irAE was associated with improved overall cancer survival.	Suggests bidirectional interaction: ICIs may exacerbate periodontal inflammation, while immune-related periodontitis may reflect heightened systemic immune activation associated with better oncologic outcomes.	Ma et al., 2024 [118]
Non-small cell lung cancer (NSCLC)	Salivary microbiome analysis revealed increased levels of periodontopathic bacteria (*Treponema denticola*, *Tannerella forsythia*, *Eubacterium brachy*, *Filifactor alocis*, *Eubacterium nodatum*). Variations correlated with objective response rate, progression-free survival, and tumor PD-L1 expression.	Oral dysbiosis and specific periodontal pathogens may influence immune checkpoint inhibitor efficacy and tumor immune phenotype in NSCLC patients.	Cardona Zorrilla, 2025 [119]
Cancer (general)	Reviews evidence that chronic periodontitis is associated with upregulation of CTLA-4 signaling, contributing to impaired T-cell activation and sustained immune tolerance within periodontal tissues.	Suggests that CTLA-4-mediated immune regulation in periodontitis may intersect with systemic immune checkpoint pathways (PD-1/PD-L1), potentially influencing antitumor immunity and responses to ICIs.	Thanigaimalai et al., 2025 [120]

Abbreviations: OSCC—oral squamous cell carcinoma; PD-1—programmed cell death-1; PD-L1—programmed death ligand 1; ICIs—immune checkpoint inhibitors; irAEs—immune-related adverse events; NSCLC—non-small cell lung cancer, CTLA-4—cytotoxic T lymphocyte-associated protein 4.

## Data Availability

No new data were created or analyzed in this study. Data sharing is not applicable to this article.

## Abbreviations

The following abbreviations are used in this manuscript:

ACL	ATP-citrate lyase
APC	antigen-presenting cell
ATRA	all-trans retinoic acid
CAR-T	chimeric antigen receptor T-cell
CTLA-4	cytotoxic T lymphocyte-associated protein 4
CRP	C-reactive protein
DNA	deoxyribonucleic acid
H3K4me3	histone H3 lysine 4 trimethylation
H3K27ac	histone H3 lysine 27 acetylation
HIV	human immunodeficiency virus
HPA	hypothalamic–pituitary–adrenal
ICAM-1	intercellular adhesion molecule-1
ICIs	immune checkpoint inhibitors
IFN-γ	interferon-gamma
irAEs	immune-related adverse events
IL	interleukin
IL-1β	interleukin-1β
IL-6	interleukin-6
IL-8	interleukin-8
JAK	Janus kinase
JAK-STAT	Janus kinase—signal transducer and activator of transcription
LAG-3	lymphocyte activation gene-3
lncRNA	long non-coding RNA
LPS	lipopolysaccharide
MAPK	mitogen-activated protein kinase
MDSC	myeloid-derived suppressor cell
mROS	mitochondrial reactive oxygen species
NF-κB	nuclear factor kappa-B
NK	natural killer
NOD2	nucleotide-binding oligomerization domain-containing protein 2
NSCLC	non-small cell lung cancer
OSCC	oral squamous cell carcinoma
PD-1	programmed cell death-1
PD-L1	programmed death ligand-1
PGE_2_	prostaglandin E_2_
PRR	pattern recognition receptor
RORγt	retinoic acid-related orphan receptor gamma t
STAT3	signal transducer and activator of transcription 3
TAM	tumor-associated macrophage
TCA	tricarboxylic acid
TCR	T-cell receptor
Th17	T-helper 17
TLR	toll-like receptor
TME	tumor microenvironment
TNF-α	tumor necrosis factor-alpha
Treg	regulatory T-cell
VCAM-1	vascular cell adhesion molecule 1
VEGF	vascular endothelial growth factor
